# 3-Methyl pyruvate enhances radiosensitivity through increasing mitochondria-derived reactive oxygen species in tumor cell lines

**DOI:** 10.1093/jrr/rrt142

**Published:** 2014-01-01

**Authors:** Naoya Nishida, Hironobu Yasui, Masaki Nagane, Tohru Yamamori, Osamu Inanami

**Affiliations:** Laboratory of Radiation Biology, Department of Environmental Veterinary Sciences, Graduate School of Veterinary Medicine, Hokkaido University, Kita-18 Nishi-9, Sapporo 060-0818, Japan

**Keywords:** 3-methyl pyruvate, A549 cells, SCCVII cells, reactive oxygen species (ROS), mitochondrial metabolism, radiosensitization

## Abstract

Considerable interest has recently been focused on the special characteristics of cancer metabolism, and several drugs designed to modulate cancer metabolism have been tested as potential anticancer agents. To date, however, very few studies have been conducted to investigate the combined effects of anticancer drugs and radiotherapy. In this study, to evaluate the role of mitochondria-derived reactive oxygen species (ROS) in the radiation-induced cell death of tumor cells, we have examined the effect of 3-methyl pyruvate (MP). MP is a membrane-permeable pyruvate derivative that is capable of activating mitochondrial energy metabolism in human lung carcinoma A549 cells and murine squamous carcinoma SCCVII cells. Pretreatment with MP significantly enhanced radiation-induced cell death in both cell lines, and also led to increases in the mitochondrial membrane potential, intracellular adenosine triphosphate content, and mitochondria-derived ROS production following the exposure of the cells to X-rays. In A549 cells, MP-induced radiosensitization was completely abolished by vitamin C. In contrast, it was partially abolished in SCCVII cells. These results therefore suggest that the treatment of the cells with MP induced radiosensitization via the production of excess mitochondria-derived ROS in tumor cells.

## INTRODUCTION

There have been several reports in the literature demonstrating that the levels of intracellular reactive oxygen species (ROS) in human IM-9 multiple myeloma cells and immortalized mouse embryonic m5S cells increase several hours after cells have been irradiated [1, 2], and this increase in ROS levels has been attributed to the leakage of ROS from the mitochondria [3]. Increases in intracellular ROS levels of this type have been observed in a fibroblast cell line (NIH3T3 cells), as well as in a variety of different tumor cell lines (A549, HeLa, MKN45 and MeWo cells), following their exposure to X-ray radiation. It has been suggested that the radiation-induced upregulation of the mitochondrial electron transport chain (ETC) function and mitochondrial content could be contributing to the increases in mitochondrial ROS levels [4]. Several recent reports have also suggested that the increases in intracellular ROS in irradiated cells occur in conjunction with or as a consequence of various other pathological events, such as the bystander effect [5, 6], genetic instability [1, 7–9], and apoptosis [3, 10–13].

Taken together, these reports led us to speculate that the excess intracellular accumulation of ROS generated in the mitochondria could enhance the sensitivity of tumor cells towards radiation. It is well known, however, that energy production in most cancers is dependent on aerobic glycolysis rather than the mitochondria-related ETC system, despite the fact that oxygen is present (i.e. the Warburg effect) [14, 15]. These dormant mitochondria in cancer cells would therefore appear to act against radiosensitization via the activation of the ETC system. To overcome this limitation in cancer cells, dichloroacetate (DCA), which activates pyruvate dehydrogenase by inhibiting pyruvate dehydrogenase kinase, has been used to normalize mitochondrial function. Bonnet *et al.* [15] reported that the treatment of non-small-cell lung cancer A549 cells with DCA led to increases in intracellular adenosine triphosphate (ATP), oxygen consumption, and mitochondrial ROS, resulting in the inhibition of tumor growth and the induction of apoptosis. Similar reductions in tumor growth following DCA treatment have been reported in a number of different cancer cell lines including breast cancer [16], pancreatic [17], metastatic breast [16, 20], colon [19], prostate [20], endometrial [21], and neuroblastoma [23] cells. Moreover, Cao *et al.* [20] demonstrated that the combination of DCA with X-irradiation induced synergistic cell death in PC3 cells through the enhancement of apoptosis and G1 cell-cycle arrest. These results suggest that it may be possible to use chemical agents that target the mitochondrial metabolism to induce radiosensitization in tumor cells. The mechanism of radiosensitization associated with the use of these agents, however, remains unclear.

In this study, we have evaluated whether 3-methyl pyruvate (MP), which is a novel metabolic activating agent for mitochondria, can be used to upregulate mitochondrial functions and induce radiosensitization in human non-small-cell lung cancer A549 cells and mice squamous cell carcinoma SCCVII cells. MP is known to be highly membrane permeable because of its lipophilicity, and is a much more favorable substrate for the tricarboxylic acid (TCA) cycle than pyruvic acid [23, 24]. To examine the relationship between the level of excess mitochondrial ROS and cell death, we have also tested the effect of the post-irradiation treatment of cells with the antioxidative agent vitamin C, in terms of their clonogenic survival.

## MATERIALS AND METHODS

### Reagents

Tetramethylrhodamine methyl ester (TMRM) and MitoSOX^TM^ Red (MSR) were purchased from Invitrogen (Carlsbad, CA, USA). ATP assay kits were purchased from TOYO B-Net Co. (Tokyo, Japan). MP, vitamin C (L-ascorbic acid sodium salt), and all of the other reagents used in the current study were obtained from Wako Pure Chemical Co. (Osaka, Japan). All of the materials were used as supplied without further purification.

### Cell culture condition

Human lung carcinoma A549 cells and murine squamous carcinoma SCCVII cells were maintained in RPMI 1640 or α-MEM medium (Invitrogen) supplemented with 10% fetal bovine serum at 37°C in 5% CO_2_.

### Clonogenic survival assay

The cells were trypsinized, diluted, counted, and then seeded into 60-mm dishes at densities of 100–3000 cells/dish before being allowed to adhere in a 37°C incubator for 6 h. MP was added to the culture medium and the cells were incubated for 24 h. The cells were then washed twice with phosphate-buffered saline (PBS) and the medium replaced with fresh medium. Immediately after the replacement of the medium, the cells were X-irradiated using an X-ray generator (1.0-mm aluminum filter, 200 kVp, 20 mA, Shimadzu HF-350; Shimadzu, Kyoto, Japan) at a dose rate of 2.55 Gy/min, which was determined using Fricke's chemical dosimeter. The cells were then allowed to grow in a humidified 5% CO_2_ atmosphere at 37°C for 4–10 days before being fixed with methanol and stained with Giemsa solution (Sigma-Aldrich, St Louis, MO, USA). Colonies containing > 50 cells were scored as surviving cells. In the experiments used to examine the effect of vitamin C on the survival curve, vitamin C was added to the medium immediately after the X-irradiation (final concentration: 1 mM in A549 and 500 mM in SCCVII), and the cells were then incubated in the presence of vitamin C until fixation and staining for counting the colonies. The survival curves were then fitted to a linear–quadratic model using the Origin Pro 7 data analysis software (OriginLab Co., Northampton, MA, USA).

### Measurements of mitochondrial membrane potential and mitochondrial ROS production

TMRM and MSR were then used as fluorescent probes for the mitochondrial membrane potential [25] and mitochondrial ROS production [25, 26], respectively. Exponentially growing cells in 60-mm dishes were used. The cells were pre-incubated with 50 mM MP for 24 h, and then washed with PBS twice before being placed in fresh culture medium. X-irradiation was performed and the cells were then incubated for 6, 12 and 24 h. Following the first incubation, the cells were collected after being trypsinized and then incubated in a medium containing 20 nM TMRM or 2 µM MSR for 30 min at 37°C. The cells were washed twice with ice-cold phosphate buffered saline without calcium and magnesium ions (PBS [-]) and then resuspended in ice-cold, serum-free culture medium and analyzed using an EPICS XL flow cytometer (Beckman Coulter, Fullerton, CA, USA). The mean fluorescence intensity of each sample was normalized to that of a control sample to calculate the relative fluorescence intensities of the samples.

### Assay for intracellular ATP content

The intracellular ATP content was evaluated using an ATP assay kit (TOYO B-Net Co.) according to the manufacturer's protocol. Briefly, following the treatment of the cells with 50 mM of MP and/or 10 Gy of X-irradiation, as described above, the cells were incubated for 24 h. The cells (5 × 10^3^ cells/100 µl) were then transferred to the well of a 96-well plate and treated with an ATP assay reagent containing luciferase (100 µl). After incubating the plate for 30 min at 25°C, the chemiluminescence from each well was measured at 25°C with a luminometer (Luminescencer-JNR; ATTO, Tokyo, Japan).

### Statistical analysis

All of the current results have been expressed as the mean values ± SEM of at least three independent experiments. Statistical analyses were performed with the Student's *t*-test. The minimum level of significance was set at *P* < 0.05.

## RESULTS

### MP-induced radiosensitization in A549 cells and SCCVII cells

**Fig. 1.**
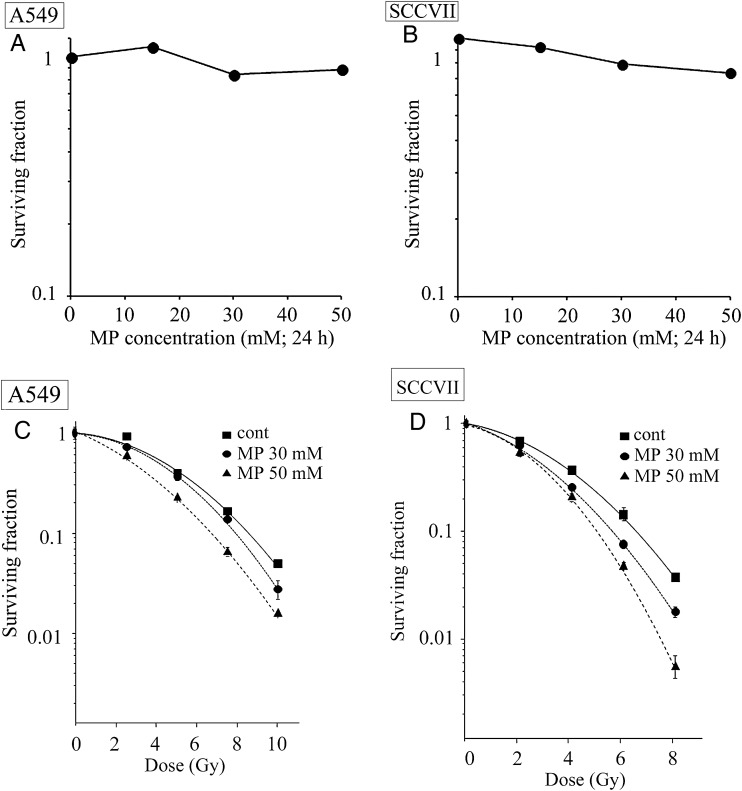
Effects of MP treatment on cytotoxicity and radiosensitivity in A549 and SCCVII cells. Clonogenic survival curves obtained from (**A**) A549 cells and (**B**) SCCVII cells treated with 15, 30 and 50 mM MP alone for 24 h. Dose–response curves for cells exposed to X-irradiation with or without the treatment of the indicated concentration of MP. After being pretreated with MP for 24 h, the cells were irradiated and assessed for any radiosensitizing effect by measuring clonogenic cell survival. (**C**) Clonogenic survival curves for A549 cells. Filled squares = X-irradiation alone, filled circles = X-irradiation + 30 mM MP, filled triangles = X-irradiation + 50 mM MP. (**D**) Clonogenic survival curves for SCCVII cells. Filled squares = X-irradiation alone, filled circles = X-irradiation + 30 mM MP, filled triangles = X-irradiation + 50 mM MP. Data are expressed as mean ± SEM.

A clonogenic survival assay was performed to examine the cytotoxicity of MP towards the A549 and SCCVII cell lines. Figure 1A and B show the cytotoxicity profiles for the A549 cells and SCCVII cells treated with various concentrations of MP for 24 h, respectively. The treatment of both cell lines with 15 mM MP did not result in any cytotoxicity. At a concentration of 30 mM, the cytotoxicity of MP was 16 ± 7% in the A549 cells and 21 ± 2% in the SCCVII cells. Further increasing the concentration of MP to 50 mM did not lead to significant changes in the cytotoxicity, with values of 12 ± 1% and 27 ± 4% in the A549 and SCCVII cells, respectively. The treatment of both cell lines with 15 mM MP did not lead to any significant radiosensitization (data not shown). In contrast, however, the pretreatment of both cell lines with 30 or 50 mM MP led to significant levels of radiosensitization in a dose-dependent manner (Fig. 1C and D). The 10% lethal dose (LD_10_) of the surviving fraction in the A549 cells was reduced from 8.80 Gy in the control to 6.75 Gy in the cells that had been treated with 50 mM MP. In the SCCVII cells, the LD_10_ was also reduced from 6.65 Gy in the control to 5.20 in the cells that had been treated with 50 mM MP. The sensitizer enhancement ratios (SER) calculated from these LD_10_ measurements were 1.30 and 1.28 in the A549 and SCCVII cells, respectively. These data indicated that the MP treatment enhanced the radiosensitivity of the tumor cells.

### Effect of MP treatment on mitochondrial membrane potential in A549 cells and SCCVII cells exposed to X-rays

**Fig. 2.**
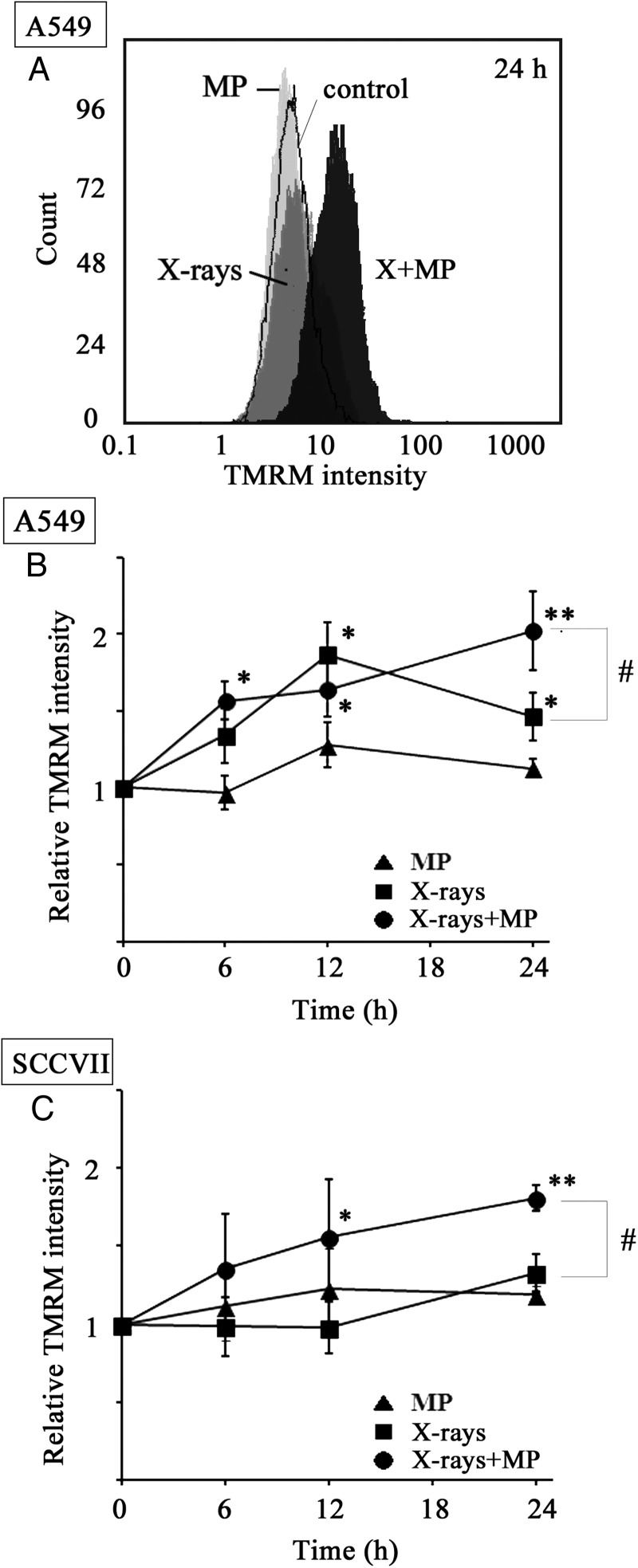
Mitochondrial membrane potentials in A549 and SCCVII cells treated with MP, X-irradiation, and a combination of X-irradiation and MP. (**A**) Typical flow cytometric profiles of the mitochondrial membrane potential in A549 cells treated with or without MP followed by X-irradiation for 24 h. White = control, light gray = 50 mM MP, dark gray = 10 Gy of X-irradiation, black = 10 Gy of X-irradiation + 50 mM MP. Time-courses of mitochondrial membrane potential obtained from (**B**) A549 cells, and (**C**) SCCVII cells treated with or without 50 mM MP followed by X-irradiation. The cells were incubated for 6, 12 and 24 h after X-irradiation and their mitochondrial membrane potential examined by flow cytometry with MSR. Each point represents the mean ± SEM of the relative value against the fluorescence mean intensity without incubation (0 h). Data are expressed as mean ± SEM of three independent experiments. The asterisk characters *(*P* < 0.05) and **(*P* < 0.01) indicate significant difference from the value without incubation time (0 h). The sharp character # (*P* < 0.05) indicates the significant difference between the MP and X-irradiation + MP.

To assess the mitochondrial function, the mitochondrial membrane potentials of the two cell lines were evaluated using TMRM as a fluorescent probe following their treatment with a combination of X-irradiation and MP [27]. Figure 2A shows the typical flow cytometric profiles obtained from the A549 cells 24 h after their treatment with either X-irradiation (10 Gy), MP (50 mM) or the combination of X-irradiation (10 Gy) and MP (50 mM). In comparison with the control, the treatment with MP alone did not have any impact on the TMRM fluorescence intensity, whereas treatment with X-irradiation alone led to a slight increase in the TMRM fluorescence intensity. The TMRM fluorescence intensity of the cells treated with a combination of X-irradiation and MP was significantly higher than that of the cells treated with X-irradiation alone. The time-courses of the TMRM fluorescence intensity in the A549 and SCCVII cells treated with X-irradiation, MP, or a combination of X-irradiation and MP were evaluated (Fig. 2B and C). As shown in Fig. 2B, the treatment of the cells with MP alone did not lead to an increase in the TMRM fluorescence intensity at any incubation time, whereas a significant increase in the TMRM fluorescence intensity was observed in the A549 cells at 12 and 24 h after their treatment with only X-irradiation. Furthermore, a significant increase was observed in the TMRM fluorescence intensity of the A549 cells that had been treated with a combination of X-irradiation and MP only 6 h after the combination treatment, and the TMRM fluorescence intensity in the same cells 24 h after this combination treatment was significantly higher than the intensity observed in the same cell line at the same time-point following X-irradiation only. For the SCCVII cell line (Fig. 2C), there was no significant change in the TMRM fluorescence intensity following the treatment of the cells with either MP or X-irradiation at any incubation time, although the increased tendency at 24 h after X-irradiation was observed. In the SCCVII cells treated with a combination of X-irradiation and MP significant increases in TMRM fluorescence intensity were observed at 12 and 24 h. These results suggested that the combination of MP treatment with X-irradiation increased the mitochondrial membrane potential in the A549 and SCCVII cells.

### Effect of MP treatment on intracellular ATP content in A549 cells and SCCVII cells exposed to X-rays

**Fig. 3.**
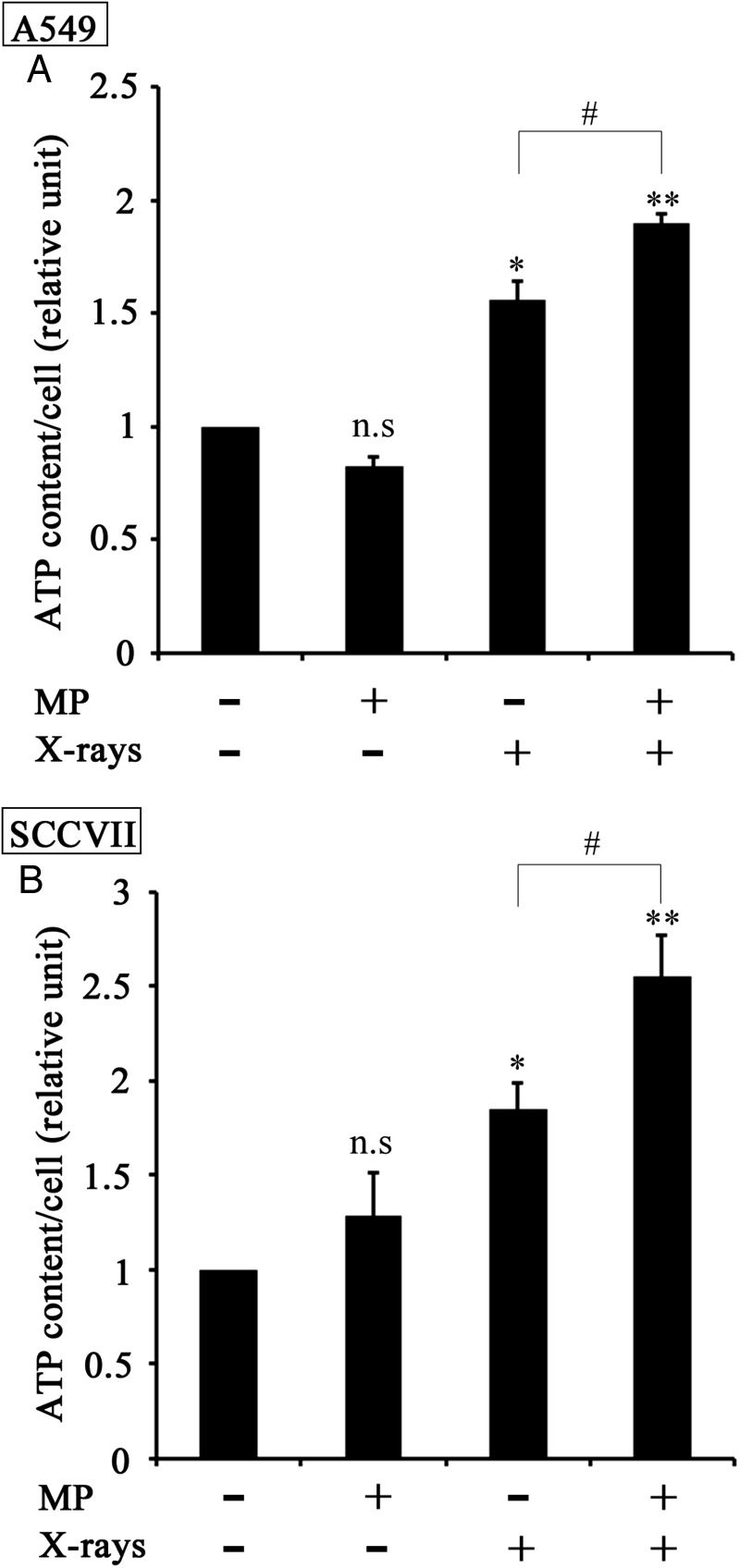
Intracellular ATP contents of the A549 and SCCVII cells treated with MP, X-irradiation, and a combination of X-irradiation and MP. The relative ATP content of (**A**) A549 cells and (**B**) SCCVII cells at 24 h after treatment with 50 mM MP, 10 Gy of X-irradiation, and 10 Gy of X-irradiation + 50 mM MP. The cells were collected and their intracellular ATP content measured as described in the text. The amounts of ATP were normalized relative to the untreated cells. All of these data are expressed as the mean ± SEM of three independent experiments. The asterisk characters *(*P* < 0.05) and **(*P* < 0.01) indicate significant difference from control. The sharp character # (*P* < 0.05) indicates significant difference between X-irradiation and X-irradiation + MP.

To determine whether the increase in mitochondrial membrane potential resulting from the combination of MP with X-irradiation led to the activation of mitochondrial energy production, we measured the intracellular ATP contents of both cell lines 24 h after their treatment with 50 mM MP or 10 Gy of X-irradiation alone, as well as after the combination of both treatments (Fig. 3). As shown, in comparison with the control, the treatment with MP alone had no effect on the intracellular ATP content, whereas X-irradiation induced a significant increase in the intracellular ATP content in A549 and SCCVII cells. Furthermore, the intracellular ATP content was significantly higher in the cells that had been treated with a combination of X-irradiation and MP than those that had been treated with X-irradiation alone in both cell lines. These results suggested that the treatment of the cells with MP facilitated the production of mitochondrial ATP in the X-irradiated tumor cells.

### Effect of MP treatment on mitochondrial ROS production in A549 cells and SCCVII cells exposed to X-rays

**Fig. 4.**
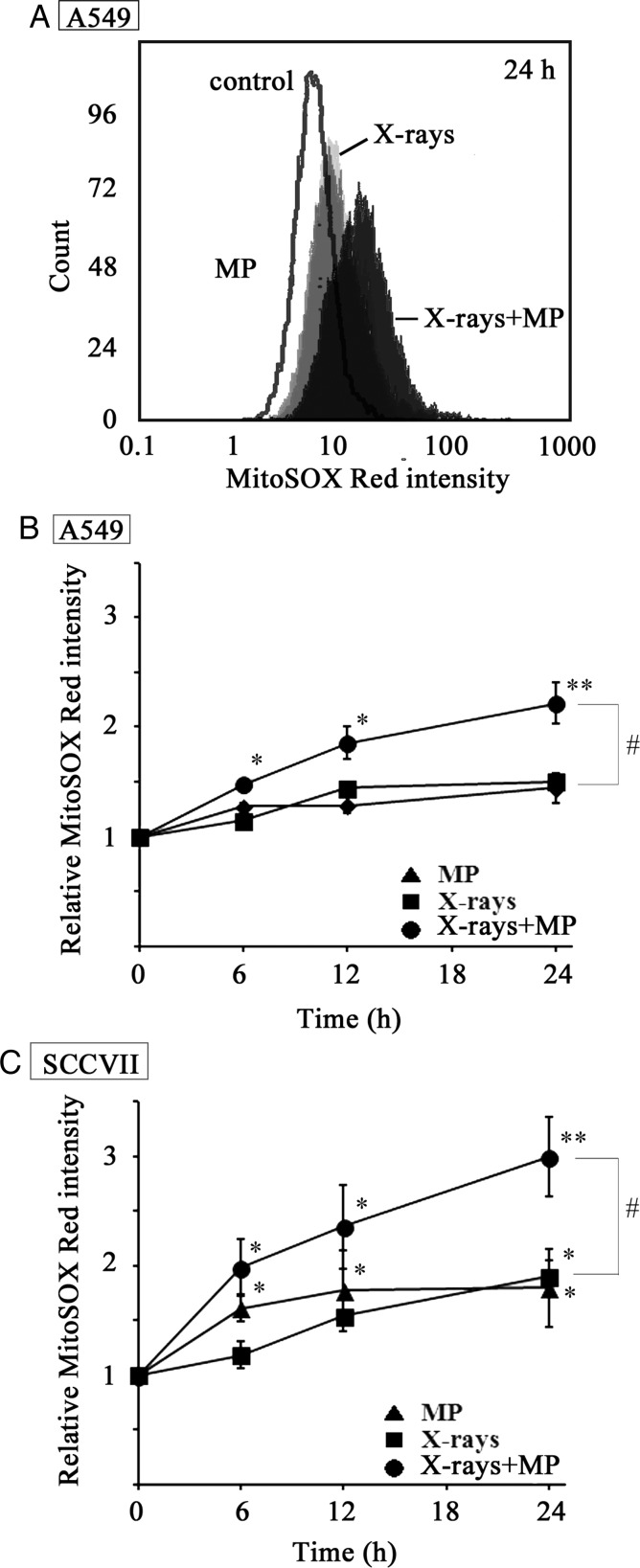
Mitochondrial ROS production in A549 and SCCVII cells treated with MP, X-irradiation, and a combination of X-irradiation and MP. (**A**) Typical flow cytometric profiles of mitochondrial ROS in A549 cells treated with or without MP followed by X-irradiation for 24 h. White = control, light gray = 50mM MP, dark gray = 10 Gy of X-irradiation, black = 10 Gy of X-irradiation + 50 mM MP. Time-courses of mitochondrial ROS production obtained from (**B**) A549 cells and (**C**) SCCVII cells treated with or without MP followed by X-irradiation. The cells were incubated for 6, 12 and 24 h after X-irradiation and their mitochondrial ROS production was examined by flow cytometry with MSR. Each point represents the mean ± SEM of the relative value against the fluorescence mean intensity without incubation (0 h). All of these data are expressed as the mean ± SEM of three experiments. The asterisk characters *(*P* < 0.05) and **(*P* < 0.01) indicate significant difference from the value without incubation time (0 h). The sharp character # (*P* < 0.05) indicates significant difference between MP and X-irradiation + MP.

Given that excessive upregulation of the mitochondrial energy metabolism is widely believed to cause the leakage of electrons from the mitochondrial ETC, which leads in turn to the production of superoxide anion radicals (O_2_^.-^) via the one-electron reduction of molecular oxygen [26], we measured the levels of O_2_^−^ and any related ROS resulting from the mitochondria in the A549 and SCCVII cell lines using the fluorescent probe MSR. Figure 4A shows the typical flow cytometric profiles obtained from A549 cells 24 h after their treatment with either 50 mM of MP or 10 Gy of X-irradiation, as well as after the combination of the two treatments. The MSR fluorescence intensity of the A549 cells treated with the combination of X-ray irradiation and MP was significantly higher than that observed in the cells treated with either X-irradiation only or MP only. Figure 4B and C show the time-courses of ROS production from the mitochondria in the A549 and SCCVII cells that had been subjected to different treatment conditions, respectively. The level of mitochondrial ROS production in the cells treated with the combination of X-irradiation and MP was significantly higher in both cell lines compared with cells that had been treated with X-irradiation alone. These results suggested that the combination of X-irradiation with MP excessively induced the activation of the mitochondrial metabolism, leading to the production of mitochondrial ROS in tumor cells.

### Effect of an antioxidant, vitamin C, on the MP-induced radiosensitization in A549 cells and SCCVII cells

**Fig. 5.**
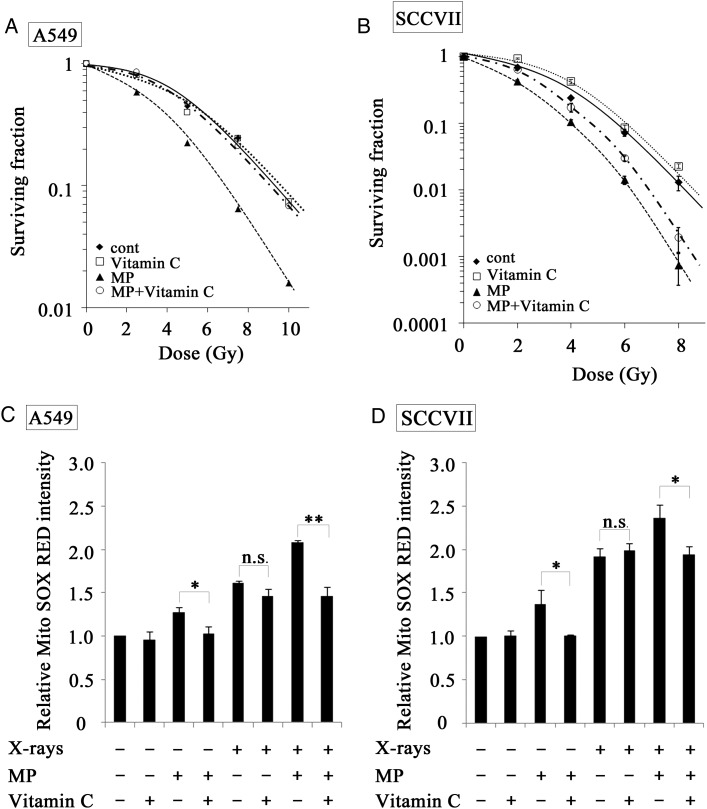
Effect of vitamin C on MP-induced radiosensitization and ROS production in A549 and SCCVII cells. Dose–response curves for (**A**) A549 cells and (**B**) SCCVII cells exposed to X-ray irradiation, with or without the treatment of 50 mM MP. After being treated with 50 mM MP for 24 h, the cells were irradiated and immediately treated with vitamin C for 4–10 days prior to a clonogenic cell survival assay, as described in the text. The concentration of vitamin C used was 1 mM for the A549 cells and 500 mM for the SCCVII cells. Filled diamond = X-irradiation alone, open squares = vitamin C + X-ray irradiation, filled triangles = X-ray irradiation + MP, open circles = X-ray irradiation + MP + vitamin C. Mitochondrial ROS production calculated from flow cytometry of (**C**) A549 and (**D**) SCCVII cells treated with 50 mM MP, 10 Gy of X-irradiation, and 10 Gy of X-irradiation + 50 mM MP for 24 h without or with vitamin C (1mM for A549 cells and 500 mM for the SCCVII, respectively). The cells were incubated for 24 h after X-irradiation and their mitochondrial ROS production were examined by flow cytometry with MSR. Each value is the mean ± SEM of three experiments. The asterisk characters *(*P* < 0.05) and **(*P* < 0.01) indicate significant difference from the value without vitamin C.

To develop a greater understanding of the role of MP-induced ROS production in the MP-induced radiosensitization, we investigated the effect of the antioxidant vitamin C on the MP-induced radiosensitization. A549 cells were treated with vitamin C after X-irradiation and the reproductive cell death was then evaluated using a clonogenic assay. As shown in Fig. 5A, the treatment with vitamin C did not have any impact on the clonogenic survival curve in the X-irradiated A549 cells that had not been treated with MP, and completely abolished MP-induced radiosensitization. In contrast, the treatment of the SCCVII cells with vitamin C only partially inhibited the MP-induced radiosensitization (Fig. 5B). Next, to confirm that mitochondrial ROS production in tumor cells X-irradiated in the presence of MP was attenuated by vitamin C treatment, we measured the levels of O_2_^−^ and any related ROS resulting from the mitochondria in the A549 cells (Fig. 5C) and SCCVII cells (Fig. 5D) using the fluorescent probe MSR, respectively. Treatment with vitamin C significantly inhibited mitochondrial ROS production in both tumor cell lines treated either with MP alone or with the combination of MP and X-irradiation. However, X-ray-induced increase in mitochondrial ROS was not attenuated by vitamin C treatment. These results suggested that the MP-induced radiosensitization occurred, at least in part, as a consequence of an increase in the production of mitochondrial ROS.

## DISCUSSION

Considerable levels of attention have recently been focused on the characteristics of cancer energy metabolism, where the majority of cancers are more dependent on aerobic glycolysis than the mitochondria-related ETC system, even in the presence of oxygen (i.e. the Warburg effect) [14]. Several drugs, such as DCA [14–22, 27, 29] and 2-deoxy-glucose [28, 29], that can be used to modulate cancer metabolism have been tested as potential anticancer agents. In this study, we have used MP as a modification agent for the mitochondria energy metabolism in tumor cells exposed to X-rays, because MP has been shown to be a substrate of the TCA cycle in the mitochondria of pancreatic β cells [23]. As shown in Figs. 2, 3 and 4, the pretreatment of cells with MP led to significant increases in the mitochondrial membrane potential, intracellular ATP content, and mitochondria-derived ROS in two X-irradiated tumor cell lines (i.e. A549 and SCCVII cells). Interestingly, the MP treatment alone did not always induce increase in these mitochondria-related parameters in A549 cells and SCCVII cells. The reason for these phenomena could not be explained definitively at the present stage, however, we speculate that not only MP as an energy substrate but also unknown factors induced by X-irradiation may be required for the energy shift from glycolysis to mitochondria oxidative phosphorylation in tumor cells. In addition, we also observed that the SER estimated from the LD_10_ of survival curves in Chinese hamster V79 lung fibroblast cell line (as a non-tumor cell line) was ∼ 1.05 (data not shown), and this MP-induced radiosensitization effect was very smaller than those effects seen in tumor cell lines (1.30 for A549 and 1.28 for SCCVII). This suggests that MP is a useful agent for activating mitochondrial energy metabolism and inducing an energy shift from glycolysis to mitochondria oxidative phosphorylation in tumor cells exposed to X-rays.

Our previous studies demonstrated that the increase in the post-irradiated levels of ROS was caused by the leakage of ROS from the mitochondria [3], and that the increase in intracellular ROS levels following irradiation occurred as a consequence of the upregulation of the mitochondrial ETC function and mitochondrial content in a fibroblast cell line, as well as in a variety of other tumor cell lines [4]. Wang and Yi [30] and Laurent *et al.* [31] have proposed a new hypothesis, which is known as the ‘ROS threshold concept’, to further characterize the role of intracellular ROS in tumor cells [32]. According to this hypothesis, a certain level of ROS is required for cell survival, but high levels of ROS could trigger cell death, and the ROS levels in tumor cells are generally much higher than they are in normal cells. Further increasing the ROS levels using a therapeutic approach could enable ROS levels to reach a death threshold in tumor cells earlier than they would do in normal cells. Based on this hypothesis, we have designed and implemented a series of experiments in the current study to determine whether intracellular ROS could be enhanced to the death threshold level *in vitro* using a combination treatment of X-irradiation and the mitochondria-targeting agent MP. As expected, pretreatment with MP led to a significant increase in the radiosensitivities of two tumor cell lines, A549 and SCCVII cells, as shown in Fig. 1. Furthermore, we have shown that this MP-induced radiosensitizing effect can be attenuated by long-term post-irradiation treatment of the cells with vitamin C (as an antioxidant), indicating that the ROS levels in tumor cells exposed to both X-rays and MP reach the death threshold level, with the tumor cells being killed as a consequence of oxidative damages. However, the extent to which ROS contributes to the MP-induced radiosensitization seems to be dependent on the tumor cell type, because only a small proportion of the MP-induced radiosensitization in SCCVII cells was attenuated, even after treatment with a high concentration (500 mM) of vitamin C, whereas the MP-induced radiosensitization in A549 cells was completely inhibited by the treatment of the cells with a low concentration (1 mM) of vitamin C. These results therefore suggest that the MP-induced radiosensitization in SCCVII cells may operate via an additional unknown mechanism rather than the ‘ROS threshold concept’.

Benhar *et al.* [32, 33] recently showed that stress-activated protein kinases (SAPKs) and ROS play an important role in the apoptotic cell death of tumor cells exposed to cisplatin (CDDP), anisomycin, or ultraviolet light (UV). In these studies, transient, low-level SAPK activity and ROS promoted cell proliferation by facilitating mitogenetic signaling pathways such as AP-1, whereas persistent high levels of SAPK activity and ROS resulted in the augmentation of the apoptotic response in tumor cells. In fact, we previously demonstrated that there was an increase in SAPK activity following X-ray irradiation in human leukemia MOLT-4 cells, and that the post-irradiation treatment of these cells with Trolox, which is a water-soluble vitamin E analog, significantly inhibited the radiation-induced apoptosis [10]. Furthermore, several studies have indicated that the excessive production of ROS from the mitochondria enhances the permeability of lysosomal membranes, resulting in the release of lysosomal proteases, which can contribute to mitochondrial membrane permeabilization and the lysosomal degradation mechanism of autophagy [34, 35]. Chui *et al.* [34] reported that the use of X-irradiation in combination with arsenic trioxide, which was used as an anti-leukemia agent to target adenine nucleotide translocase in the mitochondrial membrane [36], led to an increase in the therapeutic efficacy compared with the individual treatments in PC-3 human prostate cancer cells. Furthermore, this combination treatment also enhanced ROS generation compared with the individual treatments in the same cell line, where it led to autophagy [34]. These reports led us to speculate that SAPK or autophagy, in addition to ROS, may be associated with the mechanism underlying the observed MP-induced radiosensitization in tumor cells. Further experiments to determine the precise involvement of SAPK and autophagy in MP-induced radiosensitization in tumor cells will be necessary to develop a thorough understanding of this effect.

## CONCLUSION

In summary, we have demonstrated that the combination of X-irradiation with MP induces an upregulation of mitochondrial function that leads to an increase in the mitochondrial membrane potential as well as the production of mitochondrial ROS. The resulting substantial increase in the production of ROS effectively enhances the radiosensitivity of A549 and SCCVII cells. These results suggested that MP has the potential to be used as a radiosensitizer through the activation of mitochondrial energy metabolism in tumor cells.

## FUNDING

This work was supported, in part, by Grants-in-Aid for Basic Scientific Research from the Ministry of Education, Culture, Sports, Science and Technology, Japan (No. 24659551 [O.I.], No. 23780286 [T.Y.], and Nos. 23791375 and 25861045 [H.Y.]), and the Akiyama Life Science Foundation [H.Y.].

## CONFLICT OF INTEREST

The authors have no conflict of interest directly relevant to the content of this article.
